# Synergistic Effects of Combined Flavourzyme and Floating Electrode–Dielectric Barrier Discharge Plasma on Reduction of *Escherichia coli* Biofilms in Squid (*Todarodes pacificus*)

**DOI:** 10.3390/microorganisms12061188

**Published:** 2024-06-12

**Authors:** So Hee Kim, Pantu Kumar Roy, Shin Young Park

**Affiliations:** Department of Seafood Science and Technology, Institute of Marine Industry, Gyeongsang National University, Tongyeong 53064, Republic of Korea; thgml9903@naver.com

**Keywords:** *Escherichia coli*, biofilm, flavourzyme, FE-DBD plasma, inactivation, combination

## Abstract

This study investigated the synergistic effect of combining flavourzyme, a natural enzyme, and floating electrode–dielectric barrier discharge (FE-DBD) plasma (1.1 kV, 43 kHz, N2 1.5 m/s) treatment, a non-thermal decontamination technology, against *Escherichia coli* biofilms in squid. *E. coli* (ATCC 35150 and ATCC 14301) biofilms were formed on the surface of squid and treated with different minimum inhibitory concentrations (MICs) of flavourzyme (1/8; 31.25 μL/mL, 1/4; 62.5 μL/mL, 2/4; 125 μL/mL, and 3/4 MIC; 250 μL/mL) and FE-DBD plasma (5, 10, 30, and 60 min). Independently, flavourzyme and FE-DBD plasma treatment decreased by 0.26–1.71 and 0.19–1.03 log CFU/cm^2^, respectively. The most effective synergistic combination against *E. coli* biofilms was observed at 3/4 MIC flavourzyme + 60 min FE-DBD plasma exposure, resulting in a reduction of 1.55 log CFU/cm^2^. Furthermore, the combined treatment exhibited higher efficacy in *E. coli* biofilm inactivation in squid compared to individual treatments. The pH values of the synergistic combinations were not significantly different from those of the untreated samples. The outcomes indicate that the combined treatment with flavourzyme and FE-DBD plasma can effectively provide effective control of *E. coli* biofilms without causing pH changes in squid. Therefore, our study suggests a new microbial control method for microbial safety in the seafood industry.

## 1. Introduction

Seafood, renowned for its nutritional benefits, may present a dualistic nature as a potential source of foodborne illnesses (owing to its susceptibility to marine environmental contaminants and the potential presence of pathogenic microorganisms). Additionally, contamination of seafood may occur not only in seawater but also during processing and commercialization. Moreover, seafood products spoil quicker than other foods after capturing due to biochemical decomposition and the presence of foodborne microorganisms. Bacterial contamination continues to be a problem in the food industry worldwide, and the U.S. Food and Drug Administration [1] and European Food Safety Authority [2] have reported that *Escherichia coli* and *Salmonella*, are the most common pathogens [3].

*Escherichia coli* is a facultative anaerobic Gram-negative bacterium. It was first identified in 1982 as an enteric pathogen and is a major cause of food poisoning, which can cause serious diseases, including kidney damage [4,5]. Although extensive research is currently being conducted on reducing *E. coli*, foodborne illness still occur, causing enormous losses to the food industry and economy [6]. Additionally, *E. coli* forms biofilms, sessile bacterial colonies that are difficult to eliminate owing to their protection and which sometimes make sterilization impossible by increasing their resistance to external environmental stress [7]. Several studies have reported that biofilms of *E. coli* O157:H7 formed on food and surfaces in contact with food are resistant to dry environments and disinfection [8,9].

A biofilm is a collection of microorganisms that adhere to surfaces and each other. Bacteria grow to defend themselves by inserting cells into extracellular polymeric substances (EPSs), which improves their ability to survive against antibacterial agents [10]. Biofilm-related genes regulate the dynamic processes of biofilm formations through cell attachment, EPS synthesis, microbial maturation, cell separation, and dispersion [5,10]. In addition, biofilms are highly resistant to antibacterial treatments, because EPSs are much more resistant to external stressors and defend against antibiotics and disinfectants through resistance genes [3]. According to other studies, pathogenic bacteria form biofilms on food (salmon, shrimp, crab shell surfaces) and surfaces in contact with food (stainless steel, polypropylene, glass, and rubber), thus affecting food quality and safety [11]. In addition, biofilm formation can lead to cross-contamination, posing a serious risk to public health.

Recent, research has focused on the use of enzymes (flavourzyme, amylase and DNase) as green chemicals to reduce foodborne pathogens [7,12,13]. Flavourzyme is a natural enzyme mixture consisting of seven different endopeptidases and exopeptidases (leucine aminopeptidases, dipeptidyl peptidase, neutral and alkaline proteases) and one α-amylase [14]. It is an industrial peptidase extracted from *Aspergillus oryzae* that is used to improve the flavor of foods by promoting protein hydrolysis [15,16]. Flavourzyme is more stable than other enzymes over a wide range of pH values and temperatures, and, unlike disinfectants in the food industry, it has no residual effects. Additionally, recent reports have shown that this enzyme has antibacterial properties [3,17]. Despite these advantages, research on the control of pathogenic bacteria using flavourzyme is lacking.

Plasma refers to the fourth state of matter and includes various species such as reactive species, positive and negative ions, UV photons (e.g., UV and UVC), gas atoms, free radicals, and molecules (e.g., O_2_ and N_2_), which can achieve microbial inactivation [18]. In addition, they are generally quasi-neutral because their charges are balanced on a macroscopic scale [19,20]. 

Floating electrode–dielectric barrier discharge (FE-DBD) plasma technology has emerged as a promising method of addressing biofilm-related challenges in the food industry. This innovative approach utilizes the plasma generated within a dielectric barrier, which effectively targets and disrupts biofilm formation on various food processing surfaces. FE-DBD is different from existing dielectric barrier discharge (DBD) plasma [21]. The term floating electrode implies that the metal objects are separated; unlike DBD plasma, the discharge of the second electrode is not grounded, and the discharge does not ignite without the specimen [20,22]. Research has shown that FE-DBD plasma treatment can efficiently reduce or eliminate biofilms formed by pathogenic bacteria, such as *E. coli*, *Salmonella* spp., *Listeria monocytogenes*, and others commonly encountered in food processing environments. Its mechanism of action involves the generation of reactive oxygen and nitrogen species, which exert antimicrobial effects by damaging the biofilm matrix, disrupting cellular structures, and inhibiting microbial growth. FE-DBD plasma is used in various fields (such as the treatment of cavities and skin diseases); in particular, it has a stronger sterilization effect than existing DBD plasma [23]. 

Despite these advantages, the use of plasma is still insufficient to control foodborne pathogens. Song et al. [24] reported that sea squirts were inoculated with *V. parahaemolyticus* and treated with 60 min FE-DBD plasma, resulting in reductions of 0.75 log CFU/g. Similarly, Yong et al. [25] reported that pork jerky inoculation with *S. aureus* and *B. cereus* was, respectively, reduced by 0.99 and 0.89 log CFU/g after exposure to DBD plasma for 60 min. One of the key advantages of FE-DBD plasma technology is its ability to provide effective microbial inactivation without significant adverse effects on food quality. Unlike traditional methods, such as heat treatment, FE-DBD plasma operates at relatively low temperatures, minimizing the risk of thermal degradation of sensitive food components, such as vitamins, enzymes, and flavor compounds. Additionally, the non-thermal nature of plasma treatment reduces the likelihood of inducing undesirable changes in food texture, color, and nutritional value. The results of the above studies on microorganisms’ reduction using cold plasma show that research combining chemical decontamination techniques such as flavourzyme, a natural substance, should be conducted to effectively remove biofilms.

Several studies have combined chemical and physical treatments to reduce biofilm formations. Koban et al. [26] reported that combined treatment using atmospheric pressure plasma with NaOCl and hydrogen peroxide (H_2_O_2_) was more effective than each treatment alone in reducing *Streptococcus mutans* biofilms. In addition, Cui et al. [27] found that *S. aureus* growth on surfaces in contact with food decreased significantly when cold plasma and essential oils were combined. However, no studies have assessed combined treatment with FE-DBD plasma and the natural substance flavourzyme. Accordingly, research on effectively reducing biofilms in squid is also lacking. Therefore, objectives of this study were to evaluate the synergistic effects of flavourzyme (1/8, 1/4, 2/4, and 3/4 MIC) and FE-DBD plasma (5, 10, 30, and 60 min) on the reduction of *E. coli* biofilm formed on squid. Additionally, pH values of squid in the combination of FE-DBD plasma and flavourzyme treatment were analyzed.

## 2. Materials and Methods

### 2.1. Bacterial Strain, Culture and Growth Condition

*E. coli strains* (ATCC 35150, ATCC 14301) from the American Type Culture Collection (Manassas, VA, USA) and KCCM 12181 from the Korea Culture Center of Microorganisms (Seoul, Republic of Korea) were used for the biofilm-forming assays. The stock cultures (10^8^–10^9^ CFU/mL) were maintained in a deep freezer at −80 °C in tryptic soy broth (TSB; BD Difco, Franklin Laked, NJ, USA) containing 30% glycerol. Bacteria (100 μL) were inoculated in 10 mL TSB and incubation at 37 °C for 24 h. After 24 h, 100 μL of culture was taken and inoculated into fresh TSB and incubated at 37 °C for 20 h to optimize growth of bacteria. Then, the culture was centrifuged at 10,000 rpm at 4 °C for 10 min and washed with PBS at least twice. The final bacterial solution was diluted using peptone water (PW; BD Difco, Franklin Laked, NJ, USA) to make 10^5^ log CFU/mL [28].

### 2.2. Flavourzyme Preparation and Determination of Minimum Inhibitory Concentration (MIC)

Flavourzyme (protease from *A. oryzae*) was acquired from Sigma-Aldrich (St. Louis, MO, USA). The MIC test was performed in a previous study with slight modifications [28]. The MIC of flavourzyme against *E. coli* was determined using a two-fold serial dilution approach with TSB. Briefly, 100 μL of serially diluted flavourzyme and 100 μL of bacterial suspension (10^5^ CFU/mL) were mixed in 96-well plates (Corning Incorporated, Inc., Corning, NY, USA), and the total amount per cell was 200 μL. Then, the plate was cultured in an incubator (Vision Scientific, VS-8480, Gyeongsan, Republic of Korea) at 37 °C for 24 h, and the MIC was visually observed to confirm the lowest concentration of the flavourzyme that inhibits bacterial growth. Each bacterium was grown at 37 °C for 24 h using 1/8, 1/4, 2/4, and 3/4 MIC of flavourzyme, and the bacterial colony was counted for measuring the log CFU/mL.

### 2.3. Sample Preparation and Biofilm Formation

The squid (*Todarodes pacificus*) was purchased from online market, and upon arrival, it was immediately washed with distilled water (DW) and then used in the experiment. For biofilm experiments, the sample was cut into 2 × 2 × 0.5 cm coupons with a sterilized knife [29] and dried thoroughly on each side on a clean bench (15 min). The biofilm formation process was performed as in a previous study with slight modification [3,28]. In this study, the MIC was confirmed to be 250 μL/mL, and MIC concentrations of 0, 1/8, 1/4, 2/4, 3/4 MIC were used. Although this did not kill the bacteria, its likely affected virulence factors. The appropriate flavourzyme concentration was added to the sample, TSB, and 100 μL of *E. coli* suspension (10^5^ CFU/mL) in a 50 mL Falcon tube; the resulting mixture was vortexed with a vortex mixer (Scientific Industries, Bohemia, NY, USA) and stored at 10 °C for 7 days.

### 2.4. FE-DBD Plasma Treatment

**Figure 1:**
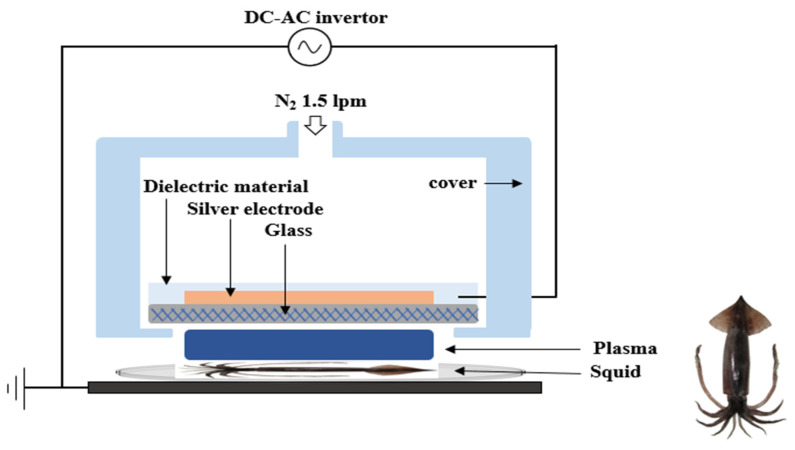
FE-DBD schematics for plasma treatment.

After forming a biofilm on the squid coupons, FE-DBD plasma treatment was performed, and the FE-DBD plasma device was delivered from the Plasma Biomedicine Institute (Plasma Bioscience Research Center, Seoul, Republic of Korea). As shown in Figure 1, the FE-DBD plasma was screen-printed with a dielectric consisting of a 10 μm thick silver electrode and 100 μm thick SiO_2_ as a high-voltage electrode on the inside of the device and a 0.7 mm thick dielectric on the outside. The operating voltage was supplied by an inventor generating a 47 kHz sine wave with an amplitude of 2.8 kV, and a nitrogen flow rate of 1.5 L was used to generate plasma between the surface of the sample, which served as a virtual ground, and the glass below the electrical electrode. All processing was performed with a 3 mm gap between samples of plasma emission electrodes. Two probes from Tektronix—one for high voltage (P6015A) and one for pickup (P6021A)—were used to monitor the electrical voltage and current of FE-DBD, respectively. The electrical power was measured at 0.55 W, the peak discharge at 16 mA, and the plasma discharge at 1 kV. The current was 13.5 mA, and the root mean square (RMS) voltage was 2.0 kV [19]. In this study, squid coupons were placed in Petri dishes and treated for 5, 10, 30, and 60 min.

### 2.5. Combination Treatment and the Synergistic Reduction Effect Analysis 

The combination treatment method and the resulting synergy effect calculation were conducted according to the study by Koivunen and Heinonen-Tanski [30]. The treatment effect was confirmed by comparing the amount of biofilm reduction after single and combined treatments with flavourzyme and FE-DBD plasma. The synergistic reduction effect value of the two treatments combined was calculated using the following equation:

Synergistic reduction value = A − (B + C).

A is the reduction value of the flavourzyme/FE-DBD plasma combination treatment, B is the reduction value of the flavourzyme treatment alone, and C is the reduction value of the FE-DBD plasma treatment alone. As a result of this equation, positive and negative values represent synergistic and antagonistic effects, respectively.

### 2.6. Biofilm Detachment Process

This process was performed with slight modification from previous studies [10,28]. After formation of biofilm, the coupon was washed with DW to remove bacteria attached to the surface of the squid. Then, the coupon was placed in a 50 mL conical tube containing 15 sterilized glass beads and 10 mL of PW and vortexed for 1 min. Then, 100 μL of this bacterial suspension was serially diluted and inoculated onto tryptic soy agar (TSA; BD Difco, Franklin Laked, NJ, USA) plates. The plate was incubated at 37 °C for 24 h, and the number of colonies was counted; the results were expressed as log CFU/cm^2^.

### 2.7. pH Value Measurement

The pH was measured to evaluate changes in squid quality due to the combined treatment with flavourzyme and FE-DBD plasma. All samples for each treatment condition were conducted in triplicate. After treatment with flavourzyme and FE-DBD plasma, 9 mL of DW was added to 1 g of squid, mixed for 5 min at room temperature, and filtered using Whatman paper (Whatman Inc., Piscataway, NJ, USA); pH values were measured using a pH meter (HI5521, Hanna Instrument Inc., Woonsocket, RI, USA).

### 2.8. Statistical Analysis with a Method to Check Normal Distribution

All data analyses were performed three times and results represented as mean ± standard deviation. Statistical analysis was performed using a one-way analysis of variance (ANOVA) and Duncan’s multiple range test with SPSS software version 27.0. Statistical significance was considered at *p* < 0.05.

## 3. Results

### 3.1. Synergistic Reduction of E. coli Biofilm with Combined Flavourzyme and FE-DBD Plasma Treatment in Squid

The reduction in *E. coli* biofilm formation in squid was determined to evaluate the effect of the combined treatment with flavourzyme and FE-DBD plasma (Table 1). The initial concentration of *E. coli* biofilm formed in squid was 6.94 log CFU/cm^2^. When treated alone at concentrations of 1/8, 1/4, 2/4, and 3/4 MIC flavourzyme, it was significantly decreased (*p* < 0.05) to 0.19, 0.68, 0.72, and 1.03 log CFU/cm^2^, respectively. FE-DBD plasma treatment alone for 5, 10, 30, and 60 min caused a decrease (*p* < 0.05) of 0.26, 0.54, 1.15, and 1.71 log CFU/cm^2^, respectively. The effects of flavourzyme and FE-DBD plasma treatments tended to increase with increasing concentration and duration of exposure. Following the combined treatment with flavourzyme and FE-DBD plasma, *E. coli* biofilm levels decreased to the range of 0.56–4.29 log CFU/cm^2^. Notably, all combination treatments led to reductions exceeding 1 log CFU/cm^2^, except for specific instances(1/8 MIC flavourzyme + 5 min FE-DBD plasma (0.56 log CFU/cm^2^), 1/8 MIC flavourzyme + 10 min FE-DBD plasma (0.77 log CFU/cm^2^), and 1/4 MIC flavourzyme + 5 min FE-DBD plasma (0.79 log CFU/cm^2^)). Moreover, when squid were treated with a combination of 3/4 MIC flavourzyme (at maximum concentration) and FE-DBD plasma for 60 min (maximum treatment duration), *E. coli* biofilms notably decreased by up to 4.29 log CFU/cm^2^. These experimental findings underscore the efficacy of the combined flavourzyme and FE-DBD plasma treatment in achieving superior reduction of *E. coli* biofilms compared to individual treatments.

Table 2 shows the synergistic effect of combined flavourzyme and FE-DBD plasma treatments on *E. coli* biofilm reduction in squid. A synergistic effect was confirmed in most combination treatments, whereas an antagonistic effect was observed in the 1/8 MIC flavourzyme + 60 min FE-DBD plasma and 1/4 MIC flavourzyme + 30 min FE-DBD plasma combinations. Notably, the application of 3/4 MIC flavourzyme with 5–60 min of FE-DBD plasma treatment exhibited a synergistic effect exceeding 1 log CFU cm^2^, with the maximum treatment duration of 60 min yielding the highest synergy reduction value (1.55 log CFU/cm^2^). Additionally, the synergistic effect against *E. coli* biofilms was independent of flavourzyme concentration and FE-DBD plasma treatment duration. The following bullet list in Table 2 represents the highest reduction results of synergistic effect of flavourzyme and FE-DBD plasma treatment.

### 3.2. pH Value of Squid Treated with Flavourzyme and FE-DBD Plasma

The pH values of the combined treatment exhibiting a synergistic effect of approximately 1 log CFU/cm^2^ or greater were analyzed (Table 3). The pH did not differ significantly between the control and experimental groups (*p* > 0.05).

## 4. Discussion

*E. coli* is an intestinal food poisoning bacterium that exists in various external environments, such as soil, drinking water, fresh agricultural and seafood products, and food processing environments [31]. It is a significant contributor to seafood contamination, which can survive in coastal marine waters for periods ranging from 60 days to three years, suggesting that it can be present in seafood [32]. Seafood contamination with *E. coli* can occur at all stages of the production and distribution process, and outbreaks have consistently been reported. Prakasan et al. [33] detected *E. coli* in 55 seafood samples (29 finfish and 26 shellfish) purchased from an Indian market, and Ryu et al. [34] identified *E. coli* in 179 of 2663 seafood products in a wholesale and retail market in Seoul, Republic of Korea. *E. coli* can adhere to surfaces and form biofilms, which contribute to the development of diverse infections, rendering them difficult to eradicate [4]. *E. coli* biofilms are highly resistant to environmental stresses, requiring a 10–100-fold increase in decontamination time and disinfectant concentration [5,35,36].

Flavourzyme, a peptidase extracted from *A. oryzae*, is taking over as a new sanitizer, owing to the negative effects of conventional sanitizers on food quality [12]. Nahar et al. [3] reported that 600 μL/mL of flavourzyme is sufficient to completely remove *E. coli* biofilms within 1 h, and Elchinger et al. [37] confirmed that >3 U mL of flavourzyme effectively inhibited *S. aureus* and *S. epidermidis* biofilms at 6–24 h. Seven different peptidases contained in flavourzyme degrade cellular peptides in the bacterial cell wall, flagella, curli, and fimbriae, thereby inhibiting biofilm formation [12,14]. The mechanism underlying the inhibition of EPS matrix formation by enzymes such as flavourzyme involves the degradation of extracellular adhesives, which diminishes intercellular connections [12]. Previous studies have demonstrated the effect of flavourzyme on biofilm inhibition, a finding corroborated by our results, which demonstrate its ability to reduce biofilm formation on the surface of squid.

FE-DBD plasma treatment, a technique commonly used in the food processing industry, can effectively eliminate foodborne pathogens without affecting food quality [19,38]. Microbial inactivation induced by plasma is associated with the generation of reactive nitrogen species (RNS) and reactive oxygen species (ROS). These reactive species damage both nucleic acids and proteins, causing the aggregation of bacteriophages and the inactivation of double-stranded DNA, single-stranded DNA, and RNA bacteriophages [39]. Various factors affect the effectiveness of plasma treatment in inhibiting microbial growth, including the microbial species, exposure conditions, gas composition, and sample depth [19]. The FE-DBD plasma treatment involves generating a non-thermal plasma discharge between a floating electrode and a dielectric barrier. This process creates a variety of reactive species, UV radiation, and electric fields, which collectively contribute to the reduction of *E. coli* biofilms. The reactive species penetrate the biofilm matrix, reaching the embedded E. coli cells. The plasma also emits UV radiation, which can further damage bacterial DNA and proteins. The reactive species break down the EPS that constitutes the biofilm matrix. As the EPS degrades, more reactive species can penetrate deeper into the biofilm, increasing the overall efficacy. As the biofilm matrix is degraded and bacterial cells are killed, the biofilm structure weakens, leading to detachment from the squid surface. Niemira et al. [40] confirmed that treatment of *Salmonella* biofilms with cold plasma for 5, 10, and 15 s reduced the counts by 1.57, 1.82, and 2.13 log CFU/g, respectively. This difference is attributed to be due to the use of higher voltage. Modic et al. [41] effectively inactivated biofilms formed by *S. aureus* and *P. aeruginosa* using cold plasma, and their results suggested that both Gram-positive and Gram-negative bacteria could be affected. Khosravi et al. [42] exposed *S. aureus* and *E. coli* biofilms to air-based atmospheric-pressure DBD plasma for up to 4 min. The respective biofilms were reduced by up to 70% and 85%, respectively, and plasma exposure caused significant inactivation of both biofilms. Czapka et al. [43] investigated the efficiency of atmospheric-pressure non-thermal plasma decontamination of *E. coli* biofilms and found that only 66.7% of the cells were killed. In addition, a lethal effect on *E. coli* was observed after 300 s; however, further removal of the dead cell layer was required. Roy et al. [44] reported bacterial reduction (1.25 log CFU/g) in salmon fillets against *L. monocytogenes* using non-thermal plasma, and the quality of smoked salmon was not altered. Dead cells prevent active plasma species from penetrating deeply into the biofilm, thereby reducing the attenuation efficacy. Therefore, to effectively remove microbial biofilms without affecting food quality, recent studies have focused on combined treatments, which can be used in the food industry to reduce microbial contamination of food and surfaces in contact with food. Additionally, if combined treatment achieves a synergistic effect, it can be considered a useful microbial decontamination technology. Jeon et al. [45] reported that combined treatment with mild heat and DBD plasma to inactivate *Bacillus cereus* in red pepper powder resulted in a reduction of approximately 6 log CFU/g. The impact of DBD plasma on *L. monocytogenes* mixed-culture biofilms was assessed by quantifying the biofilms on stainless steel (SS) at various time intervals (5–60 min) following treatment. The average reductions in biofilm counts after 5, 15, 30, 45, and 60 min were 0.11, 0.37, 0.65, 1.04, and 1.14 log CFU/cm^2^, respectively [24]. Moreover, FE-DBD plasma treatment offers several practical benefits to the food industry. It can be easily integrated into existing processing lines as a non-invasive and environmentally friendly solution for biofilm control. The versatility of plasma technology allows its application to a wide range of surfaces in contact with food, including stainless steel equipment, conveyor belts, packaging materials, and even delicate food products. In addition, the combination of a cold atmospheric-pressure plasma jet and H_2_O_2_ demonstrated a dramatic synergistic effect on *Enterococcus faecalis* inactivation [46].

To evaluate the suitability of combined treatment with flavourzyme and FE-DBD plasma, we measured squid pH. The results showed that this method of *E. coli* decontamination did not significantly affect the pH of squid, demonstrating the feasibility of the combined treatment. While research on flavourzyme, recognized as a natural antimicrobial agent, remains limited, our findings could alleviate concerns regarding alterations in food quality. As a non-thermal treatment method, cold plasma can exert negligible to minimal effects on the physical, chemical, nutritional, and sensory properties of many products [45]. However, interactions between RNS and ROS generated by plasma and food components can lead to carcinogenesis, destruction of essential nutrients, and alteration of protein, lipid, and carbohydrate functionalities [47]. Therefore, an appropriate treatment time that does not cause quality changes and can be supplemented through combined treatment with flavourzymes is needed. 

Combined treatment involving flavourzyme and FE-DBD plasma the exhibited a synergistic effect in reducing *E. coli* biofilms, surpassing the efficacy of the individual treatments. Notably, this combined approach did not induce any discernible changes in pH, suggesting its viability as an alternative method for biofilm eradication. FE-DBD plasma treatment is a promising strategy for biofilm management in squid processing. Squid surfaces prone to biofilm formation can be treated with plasma to prevent or reduce microbial attachment and biofilm development. This intervention will enhance food safety by mitigating the risk of pathogen contamination and extend the shelf life of squid products by minimizing microbial spoilage.

## 5. Conclusions

In this study, we demonstrated the effective antibacterial activity of flavourzyme and FE-DBD plasma against *E. coli* biofilms on squid. The combination treatment decreased the number of bacterial cells significantly (*p* < 0.05) more than the single treatments. The synergistic effect was decreased by more than 1 log (90%) from 3/4 MIC flavourzyme + 5 min FE-DBD plasma, suggesting that it is an effective way of removing biofilms without causing a pH change. These results indicate that combination treatment is a viable approach to improving the microbial safety of seafood. Further assessment of the nutritional and sensory aspects of the combined treatments, excluding pH, is required to further improve safety and food quality after use of the proposed method for controlling *E. coli* biofilms in foods.

## Figures and Tables

**Table 1:** Reduction of *E. coli* biofilms in squid after combined treatment with flavourzyme and FE-DBD plasma.

Microorganisms	Flavourzyme Concentration (MIC)	FE-DBD Plasma Treatment (min)				
		0	5	10	30	60
*E. coli*	0	-	0.19 ± 0.12	0.68 ± 0.11	0.72 ± 0.07	1.03 ± 0.09
	1/8	0.26 ± 0.10	0.56 ± 0.08	0.77 ± 0.08	1.00 ± 0.01 ^j^	1.44 ± 0.14 ^i^
	1/4	0.54 ± 0.18	0.79 ± 0.01	1.08 ± 0.05 ^j^	1.11 ± 0.01 ^j^	1.92 ± 0.02 ^g^
	2/4	1.15 ± 0.11 ^j^	1.97 ± 0.05 ^g^	2.38 ± 0.04 ^f^	2.75 ± 0.21 ^e^	3.07 ± 0.14 ^d^
	3/4	1.71 ± 0.13 ^h^	3.01 ± 0.19 ^d^	3.38 ± 0.15 ^c^	3.90 ± 0.05 ^b^	4.29 ± 0.19 ^a^

Data represented as means with standard deviations (three samples/treatment). The gray boxes are combination treatments with >1 log reduction; different letters (a–j) represent significant differences (*p* < 0.05) were found via Duncan’s multiple-range test with 5% probability.

**Table 2:** Synergistic and antagonistic effect of *E. coli* biofilms in squid after combined treatment with flavourzyme and FE-DBD plasma.

Microorganisms	Flavourzyme Concentration (MIC)	FE-DBD Plasma Treatment (min)			
		5	10	30	60
*E. coli*	1/8	0.11 ± 0.03	0.02 ± 0.08	0.02 ± 0.04	−0.59 ± 0.06
	1/4	0.06 ± 0.13	0.05 ± 0.18	−0.15 ± 0.17	0.35 ± 0.14
	2/4	0.63 ± 0.10	0.74 ± 0.12	0.88 ± 0.03	0.89 ± 0.14
	3/4	1.11 ± 0.11 ^b^	1.18 ± 0.20 ^b^	1.47 ± 0.03 ^a^	1.55 ± 0.03 ^a^

Data represented as means with standard deviations (three samples/treatment). Synergistic effect represented as + = (reduction achieved with flavourzyme + FE-DBD plasma) − {(reduction with flavourzyme + reduction with FE-DBD plasma)}. Antagonistic effect represented as − = (reduction achieved with flavourzyme + FE-DBD plasma) − {(reduction achieved by flavourzyme + reduction by FE-DBD plasma)}. The gray boxes are combined effect >1 log reduction; different letters (a,b) represent significant differences (*p* < 0.05) were found by Duncan’s multiple-range test with 5% probability.

**Table 3:** Combined treatment with flavourzyme and FE-DBD plasma effect in the pH of squid.

	Flavourzyme Concentration (MIC)	FE-DBD Plasma Treatment (min)				
		0	5	10	30	60
pH	3/4	6.42 ± 0.19 ^NS^	6.42 ± 0.05	6.38 ± 0.03	6.36 ± 0.02	6.36 ± 0.01

NS; non-significant. Data represented as mean ± SD (three samples/treatment). Data within the same row indicate significance (*p* < 0.05) found via Duncan’s multiple-range test.

## Data Availability

Data are contained within the article.
